# Two-dimensional B$_2$C as a potential anode material for Mg-ion batteries with extremely high theoretical capacity

**DOI:** 10.1038/s41598-022-15702-9

**Published:** 2022-07-06

**Authors:** Grzegorz T. Kasprzak, Artur P. Durajski

**Affiliations:** Institute of Physics, Czestochowa University of Technology, Ave. Armii Krajowej 19, 42-200 Czestochowa, Poland

**Keywords:** Electronic properties and materials, Surfaces, interfaces and thin films

## Abstract

The development of new high-capacity anode materials using ions other than lithium as a charge carrier is one of the essential strategies in searching for next-generation high-performance rechargeable batteries. Herein, using first-principles computations, we explore a B$_2$C monolayer as a potential anode material for Mg-ion batteries. The high stability of the free-standing B$_2$C monolayer has been demonstrated via calculating the adsorption energy, phonon dispersion, and *ab-initio* molecular dynamics simulations. The metallic character of the B$_2$C monolayer, desirable from the point of view of energy storage, ensures good electronic conductivity during the battery charge/discharge process. The calculated migration energy barrier, open-circuit voltage, and theoretical specific capacity of the B$_2$C monolayer are much better than those of some other two-dimensional materials. These findings provide the B$_2$C monolayer as a potential candidate for Mg-ion battery anode material with a high theoretical specific capacity of 3187.55 mAh/g.

## Introduction

Due to the rapid growth in global demand for energy, the development of high-performance energy storage devices with high energy density is much desirable^1–3^. Currently, rechargeable batteries have attracted great attention due to their advantages like portability and reusability. Li-ion batteries (LIBs) have been developing rapidly during the past decades, and they are use in many places from portable electronic devices, such as mobile phones, and laptop computers to electric cars^4, 5^. However, the limited lithium resources in the Earth’s crust and the associated continuously increasing cost of this material lead to the high production cost of LIBs^6^. Recently, ions of various alkali metals such as sodium (Na$^+$) and potassium (K$^+$) have attracted significant research attention because of their low-cost and similar chemistry to Li$^+$. On the other side, the multivalent ions of alkali earth metals such as magnesium-ion (Mg$^{2+}$) and calcium-ion (Ca$^{2+}$) are another appropriate choice that could replaced Li$^+$ due to their relative abundance, good environmental compatibility, and greater charge densities that lead to higher theoretical capacities^7–10^. However, one of the most critical challenges for metal-ion batteries is the development of high-performance anode materials, which ensure adequate capacity, power density, charge and discharge rates, and cycle life of the battery^11^.

Many two-dimensional (2D) materials like silicene, phosphorene, transition metal dichalcogenides (TMDs), and transition-metal carbides (MXenes) have shown a high potential to be used as anode materials for alkali and alkaline earth metal ion batteries due to the unique physical/chemical properties and high surface-to-volume ratios^12–16^. Thus they would provide rapid ions migration as well as sufficient channels for ions insertion, delivering high capacity and rate performances^3^. One of the most widely studied 2D materials is graphene^17^. However, its applications in batteries are severely limited due to its relatively low capacity (372 mAh/g for LIBs)^18^. Nevertheless, graphene-like nanostructures can be used as a potential negative electrode material for rechargeable ion batteries. For instance, Liu *et al.* showed that new graphene and nitrogen monolayer, C$_3$N, is a promising material for LIBs, which exhibits low open-circuit voltage (0.12 V), high reversible capacity (840.35 mAh g$^{-1}$), fast charging/discharging rate, and good electronic conductivity^19, 20^. Jana *et al.* designed buckled graphene-like monolayer structure PC$_3$ with remarkably high theoretical capacity (1200 mAh g$^{-1}$) and an ultralow sodium diffusion barrier^21^. Good application performance in LiBs fields exhibits also PC$_6$ with the storage capacity value of 478.61 mAh g$^{-1}$ and 717.09 mAh g$^{-1}$ reached for Li atom respectively adsorbed on single and double side of PC$_6$ monolayer^22^. It was recently shown that boron and carbon 2D material named B$_2$C should find potential applications in future nanomechanics, electronics, and optoelectronics. For example, Dai *et al.*, based on first-principles calculations, reported that B$_2$C is a 2D phonon-mediated superconductor with a relatively high transition temperature of 19.2 K^23^. Recent reports show also that B$_2$C has enormous potential to be applied as anode material for LIBs/NIBs. In this case, the results reveal that B$_2$C exhibits a very high theoretical capacity and a low diffusion barrier^24, 25^.

Motivated by the above report on the excellent electrochemical performance of B$_2$C as an anode material for LIBs and NIBs, we performed first-principles simulations to investigate the properties of monolayer B$_2$C as anode material for rechargeable Mg-ion batteries.

## Results and discussion

**Figure 1:**
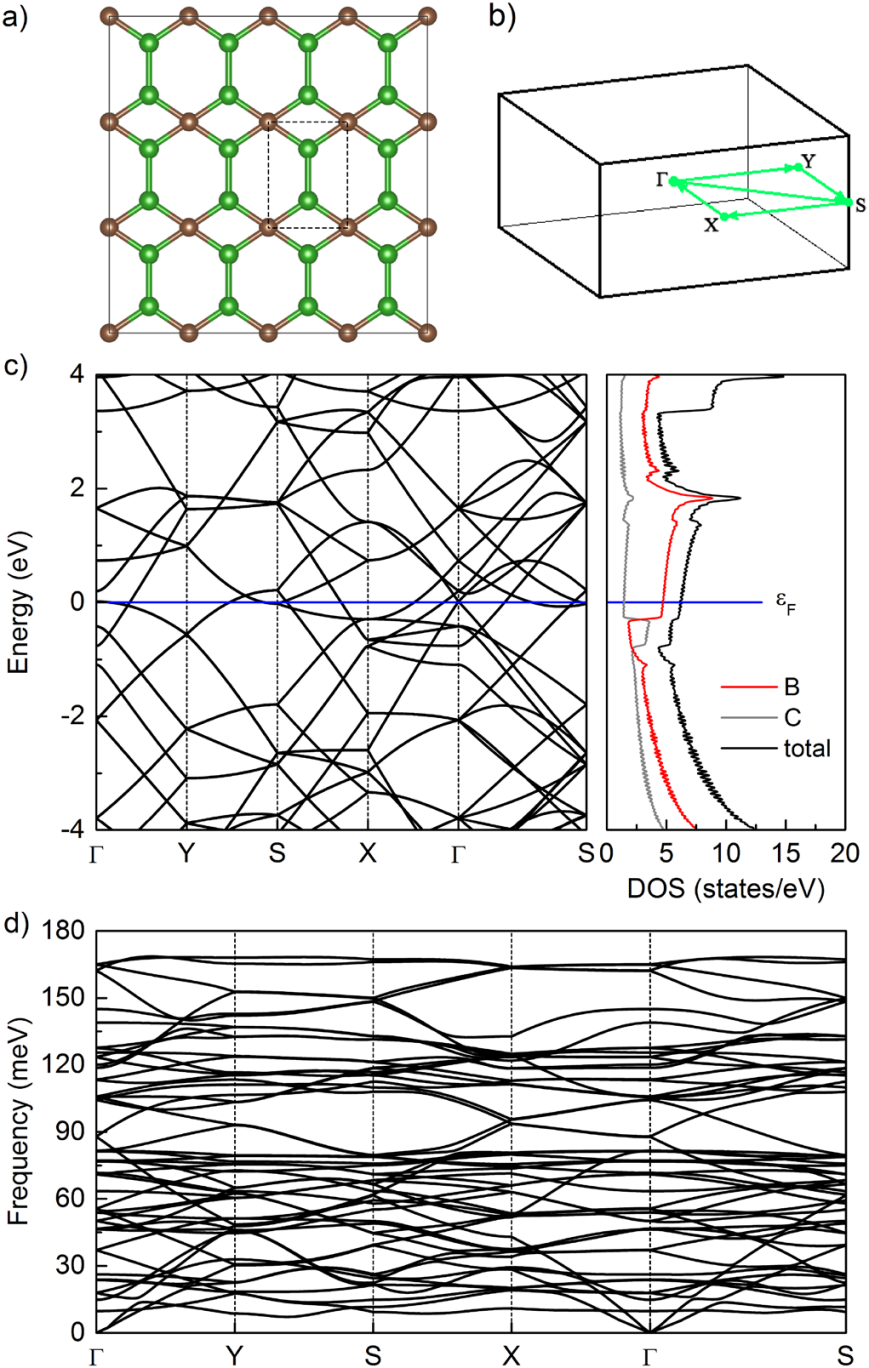
(**a**) Optimized structure of $4\times 3$ supercell of B$_2$C with the rectangle indicating the unitcell. Green for B, brown for C. (**b**) The Brillouin zone for P2mm structure with special k-point paths. (**c**) The electronic band structure of B$_2$C supercell along the $\Gamma$-Y-S-X-$\Gamma$-S high-symmetry line, together with the total and partial density of states. The Fermi level $\varepsilon _F$ is set to zero. (**d**) Phonon-dispersion relations of pristine B$_2$C monolayer.

A first-principles prediction of a new two-dimensional inorganic material, namely, the B$_2$C was reported by Wu *et al.*^26^. As shown in Fig. 1a, different from graphene, B$_2$C is composed of hexagons and rhombi with one carbon atom and two boron atoms per unit cell (the black dashed framework). We find the calculated relaxed lattice parameters of freestanding B$_2$C to be $a = 2.578$ Å and $b = 3.425$ Å, and the bond lengths of B-B and B-C are 1.556 Å and 1.683 Å, respectively, which are in good agreement with the previous studies^23, 24, 27^. The corresponding electronic band structure along high symmetry lines in the Brillouin zone (see Fig. 1b) and density of states are given in Fig. 1c, from which one can see that $4\times 3$ supercell of B$_2$C exhibits metallic nature with several bands crossing the Fermi level. The metallic character ensures good electrical conductivity which is desired for efficient anode materials. Moreover, the calculated phonon dispersion curves for B$_2$C are shown in Fig. 1. A foremost important observation is the positive frequency of all phonon modes throughout the Brillouin zone, which indicates the dynamical stability of the B$_2$C structure.

**Figure 2:**
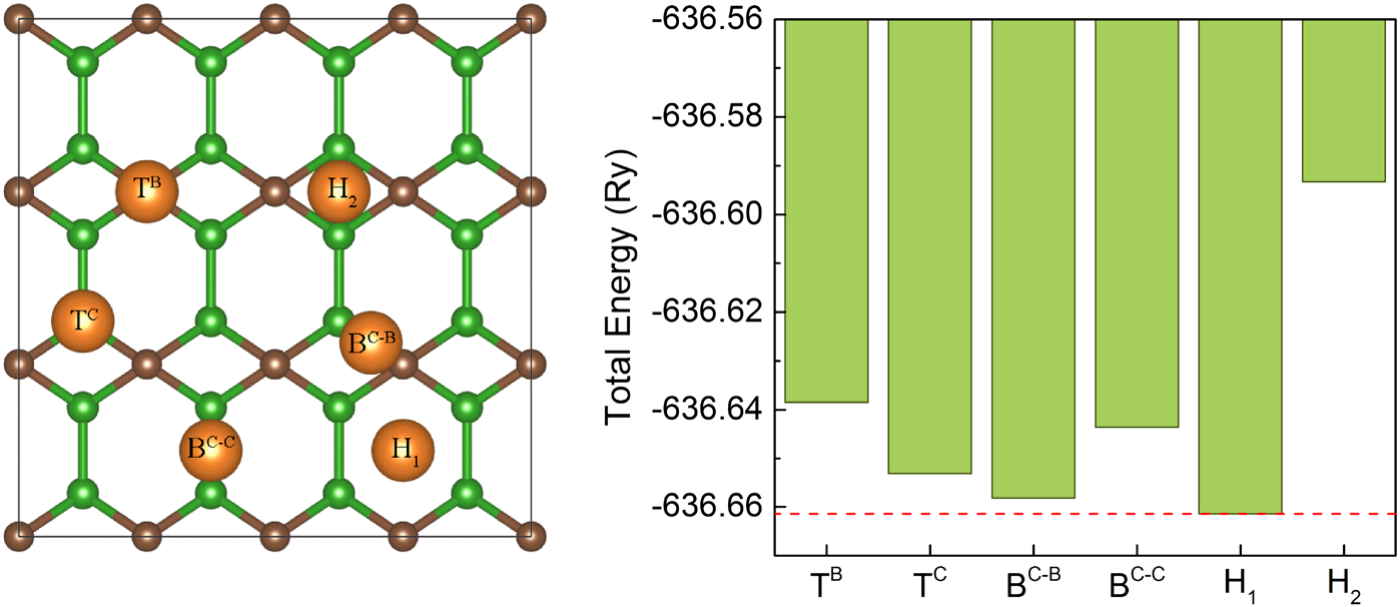
Possible adsorption sites on B$_2$C surface for Mg atom (left panel) and comparison of total energy for a single Mg atom adsorbed at different sites on B$_2$C monolayer (right panel). The red dotted line markers the values of the lowest energy.

The Mg adatom can either adsorb on a bridge site (B$^{\mathrm{C-C}}$) above a C–C bond, bridge site (B$^{\mathrm{C-B}}$) above a C–B bond, on top (T$^{\mathrm{C}}$) of a carbon site, on top (T$^{\mathrm{B}}$) of a boron site, in the hole of a hexagonal ring (H$_1$) or in the hole of rhombus (H$_2$) - see Fig. 2. For each adsorption site, a geometry optimization was performed. As we can see the energetically more favorable is H$_1$ position.

**Figure 3:**
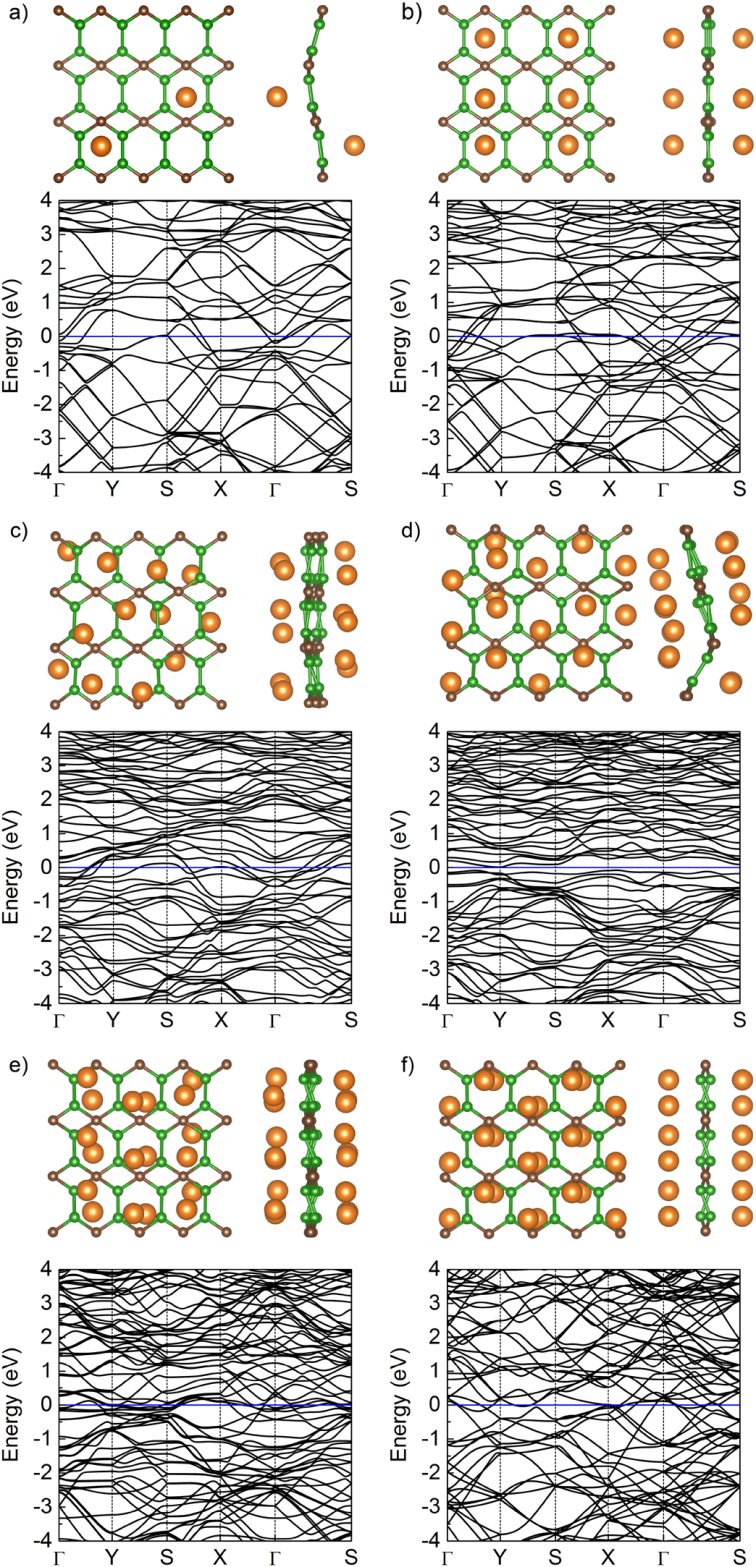
The top view and side view of relaxed structure of $4 \times 3$ supercell of B$_2$C monolayer with (**a**) 2, (**b**) 6, (**c**) 12, (**d**) 16, (**e**) 18, and (**f**) 24 adsorbed Mg atoms. The corresponding electronic band structures are presented below.

Yu *et al.*^24^ also reported that after full structural optimization the most stable adsorption site on B$_2$C surface for Li/Na is the sites above the center of the hexagonal B$_2$C ring. Starting from this most favorable position we considered a series of configurations with the chemical stoichiometry of $\mathrm Mg_{ n}B_2C$ ($n = 0.167, 0.5, 1, 1.333, 1.5, 2$) where Mg atoms were initially adsorbed onto the hollow site (H$_1$) on both-sides of B$_2$C. As a result of a structure optimization, the B$_2$C substrate is strongly modulated as shown in Fig. 3. In particular, for higher concentrations, the Mg atoms move significantly outwards from their initial hollow positions, due to their mutual Mg-Mg atoms interaction. Energetically more favorable is to maximize the distance between the Mg atoms. For this reason, the Mg atoms were moved, in the optimization process, on top of the atom or bond. However, no structural destruction was observed, and the Mg atoms were still tightly adsorbed on the B$_2$C surface. All of these observations indicate that the structures under our study are dynamically stable. From an application point o view only strong deformation of structure Mg$_{1.333}$B$_{2}$C (Fig. 3d) is undesirable because can effects the operation of the battery under experimental conditions, especially the swelling effect (volumetric expansion) during multiple charge/discharge cycles can appear.

In electrode materials, relatively large adsorption energy is important in the process of adsorbing metal ions. The average adsorption energy of Mg atoms we determined from:

1 $$\begin{aligned} E_{\mathrm{ads}}=({E_{\mathrm{Mg_{ n}}B_2C}} - E_{\mathrm{B_2C}} - nE_\mathrm{Mg})/n, \end{aligned}$$

where $E_{\mathrm{B_2C}}$ is the total energy of pristine B$_2$C monolayer; $E_{\mathrm{Mg}}$ is the energy of a single Mg atom in the bulk structure (hcp); *n* is the number of adsorbed Mg atoms, and ${E_{\mathrm{Mg_{ n}}B_2C}}$ means the total energy of B$_2$C with *n* adsorbed Mg atoms on both sides. From this definition, more negative adsorption energy means that the adsorption is more favorable and the adsorbing system is more stable. The results of the calculations of several Mg concentrations on double side configurations are collected in Table 1.

**Table 1:** The calculated average adsorption energy ($E_{\mathrm{ads}}$) and theoretical capacities (*C*) for B$_2$C with Mg adsorbed atoms.

Configuration	$E_{\mathrm{ads}}$ (eV)	*C* (mAh/g)
Mg$_{0.167}$B$_{2}$C	−0.9258	265.629
Mg$_{0.5}$B$_{2}$C	−1.0150	796.888
Mg$_{1}$B$_{2}$C	−1.4456	1593.78
Mg$_{1.333}$B$_{2}$C	−1.6179	2125.03
Mg$_{1.5}$B$_{2}$C	−1.6428	2390.66
Mg$_{2}$B$_{2}$C	−1.8376	3187.55

We noticed that in all cases the adsorption energy keeps a negative value and systematically decreases by increasing the Mg concentration. The minimum value of adsorption energy ($E_\mathrm{ads}=-1.8376$ eV) is achieved when both sides of B$_2$C are fully covered with 24 Mg atoms which correspond to the Mg$_{2}$B$_{2}$C configuration. It should be noted, that usually the average adsorption energy gradually becomes less negative with increasing metal atoms coverage, indicating weaker and weaker binding. This is primarily caused due to the enhanced repulsions between the positively charged metal adatoms. However, in the case of Mg atoms, the situation is entirely different, similar to this previously observed for the borophene sheet explored as the Mg-ion anode material^28^.

The maximum theoretical specific capacity (*C*) of B$_2$C can be computed via the following equation:

2 $$\begin{aligned} C=\frac{nzF}{\text {M}_{B_2C}}, \end{aligned}$$

where *n* is the number of adsorbed Mg atoms, *z* is the valence number ($z=2$ for Mg), *F* is the Faraday constant (26801 mAh/mol), and $\mathrm M_{B_2C}$ is the molar mass of B$_2$C substrate. The results in Table 1 show that, for the maximum Mg concentration with both-side adsorption (Mg$_{2}$B$_{2}$C), the B$_2$C monolayer could provide an extremely high capacity of 3187.55 mAh/g which is much larger compared to the storage capacities of Mg for other 2D anode materials like C$_2$N (588.4 mAh/g)^29^, VO$_2$ (815 mAh/g)^30^, phosphorene (865 mAh/g)^31^, arsenene (1430.9 mAh/g)^32^, borophene (1960 mAh/g)^33^, flat borophene films (2480 mAh/g)^28^, BC$_3$ monolayer (796 mAh/g)^34^ and close to that of BSi (2749 mAh/g)^35^.

**Figure 4:**
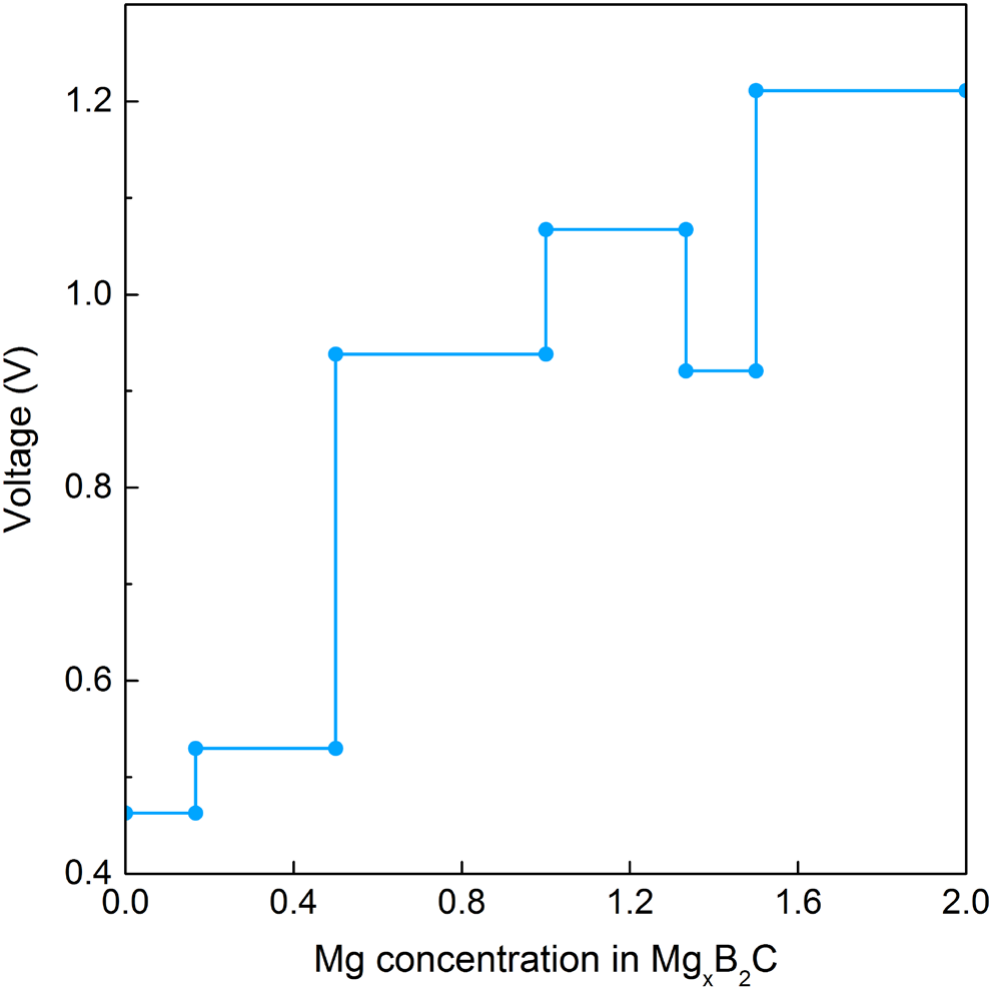
Calculated voltage profile with respect to Mg content from 0 to 2.

**Figure 5:**
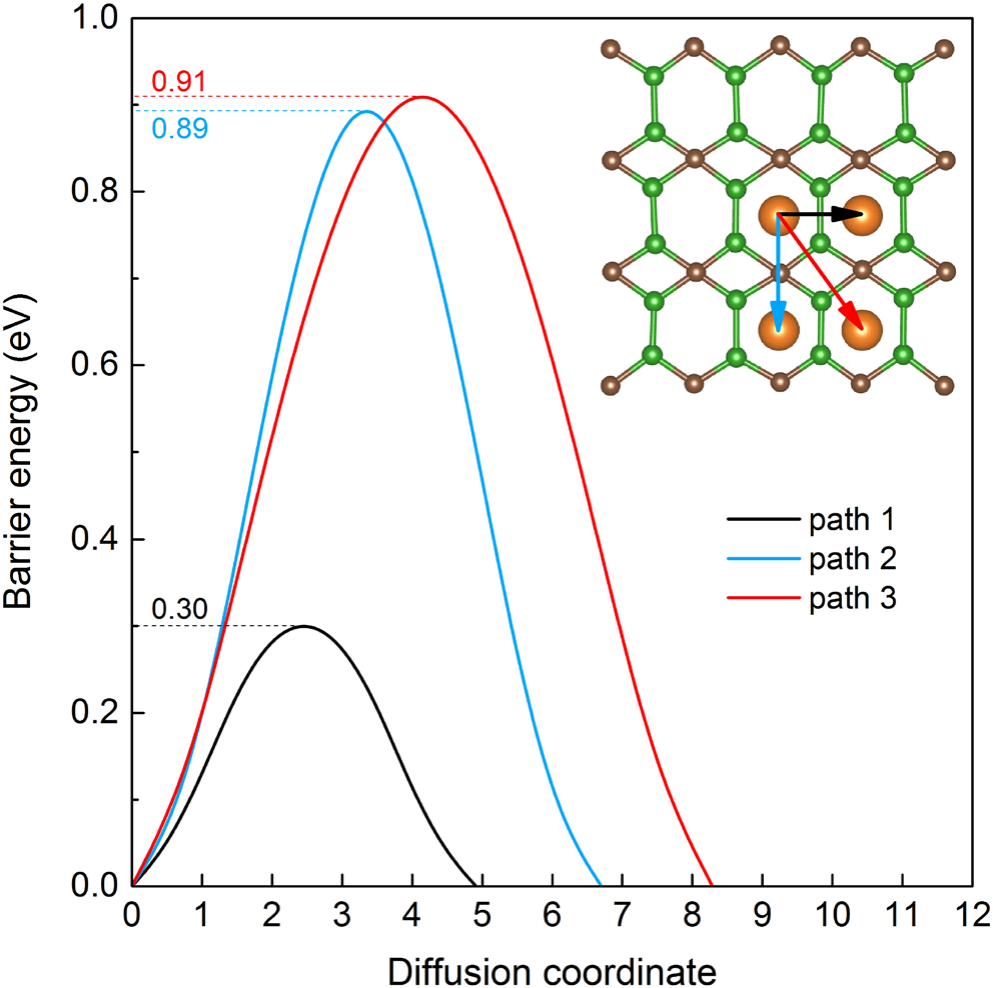
Migration barrier energies of Mg atom from the energetically stable absorption hole site to nearest hole site.

**Figure 6:**
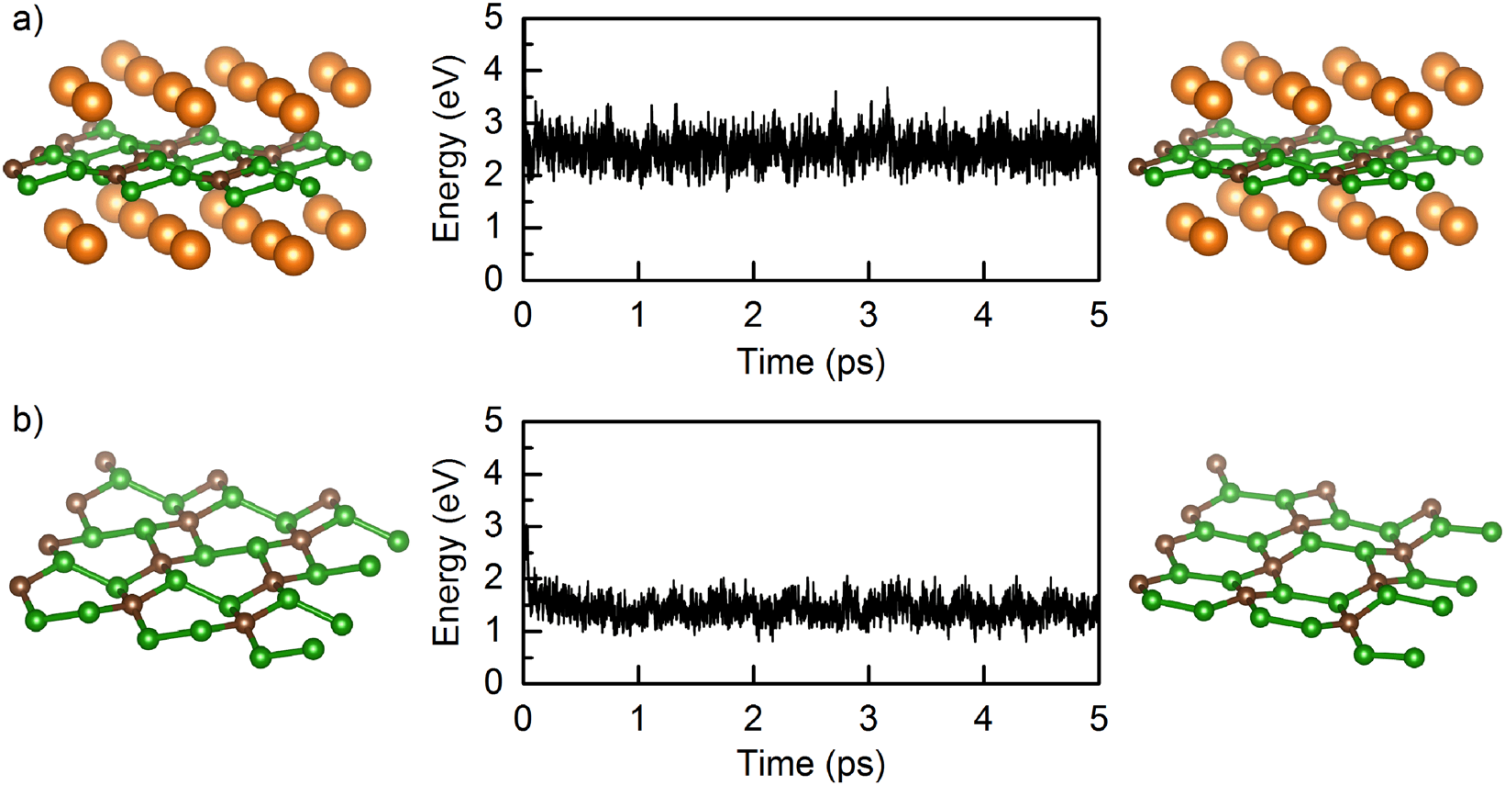
Kinetic energy fluctuation as a function of time and corresponding snapshots before and after simulation for (**a**) fully charged B$_2$C monolayer (Mg$_{2}$B$_2$C) and (**b**) fully discharged B$_2$C monolayer at 300 K.

**Figure 7:**
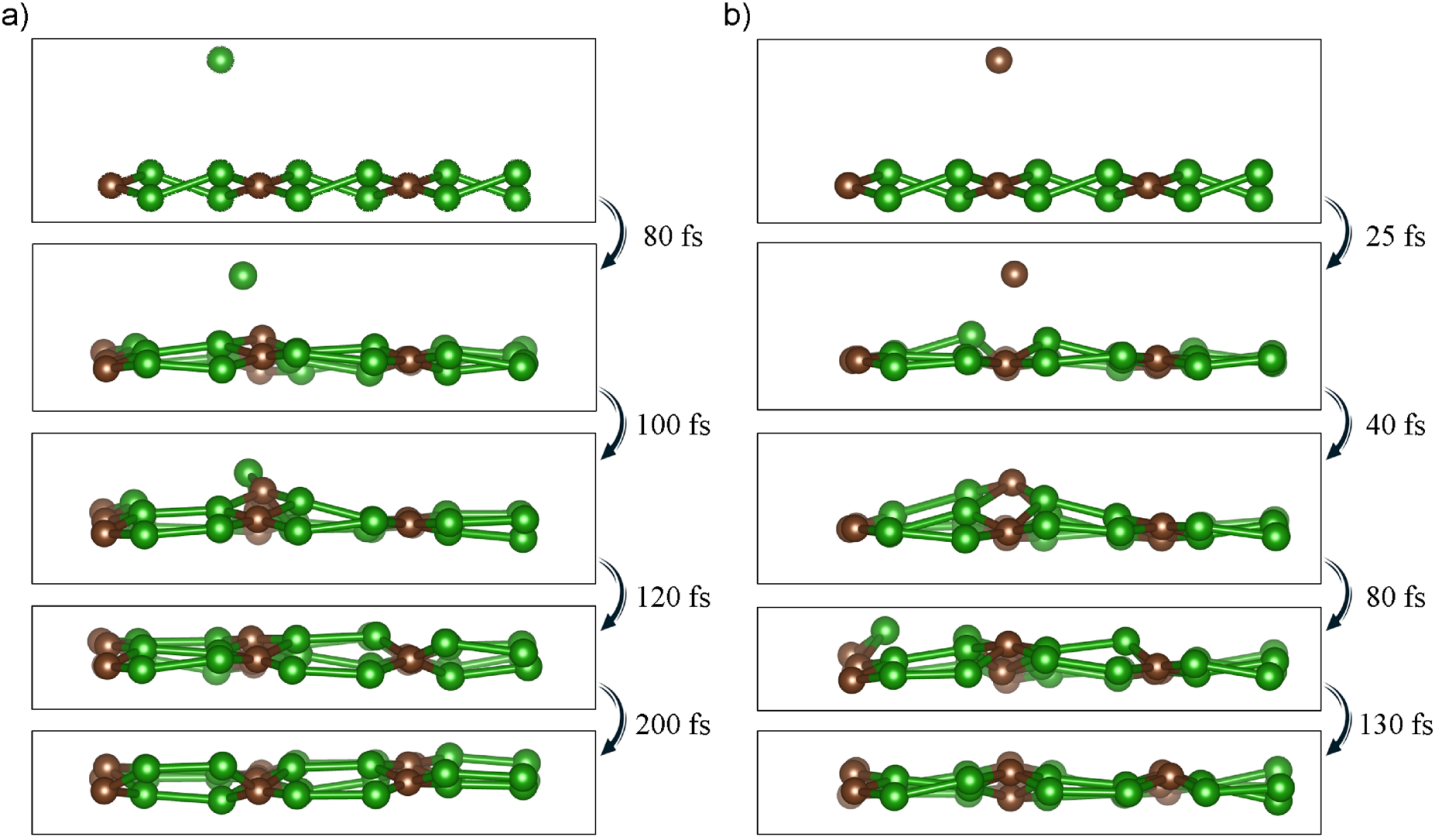
Spontaneous self-healing of B$_2$C monolayer without any external stimulus at 300 K. The point defect was introduced by extracting the one boron atom (**a**) or one carbon atom (**b**) from the surface of the material to a distance of 2.7 Å.

Open circuit voltage (OCV) is another crucial parameter to characterize the performance of metal-ion batteries. In theory, the OCV curve can be obtained by calculating the energies in the concentration range of $n_1< n < n_2$:

3 $$\begin{aligned} OCV\approx \frac{{-E_{\mathrm{Mg_{ n_2}}B_2C}}+{E_\mathrm{Mg_{ n_1}B_2C}}+(n_2-n_1)E_{\mathrm{Mg}}}{z(n_2-n_1)e}, \end{aligned}$$

where, $E_{\mathrm{Mg_{ n_2}}B_2C}$, $E_{\mathrm{Mg_{ n_1}}B_2C}$ and $E_{\mathrm{Mg}}$ are the energies of $\mathrm{Mg_{ n_2}B_2C}$, $\mathrm{Mg_{ n_1}B_2C}$ and single Mg atom in the bulk structure (hcp), respectively. Symbol *e* denotes the fundamental charge and *z* is the electronic charge of Mg ion ($z= 2$ for Mg). Figure 4 shows the voltage profiles of the B$_2$C sheet for various Mg content. It is important to note that the voltage remains positive during the whole range of coverage values (the voltage potential ranges from 0.46 to 1.21 V which is desirable for anode materials). A negative value means that the metal ion prefers to form metallic states instead of adsorbing on the anode surface^28^. Thus, the lack of negative voltage indicates that B$_2$C monolayer is suitable to be the anode materials for Mg-ion batteries. The average potential value of 0.86 V is compared to value computed for VS$_2$ monolayer (0.93 V)^36^ and is higher than that of other 2D anode materials for Li-ions, such as MoS$_2$ (0.26 V)^36^, Mo$_2$C (0.68 V)^37^, Ti$_3$C$_2$ (0.43 V)^38^. From the above findings, we conclude that B$_2$C monolayer can be considered as a potential anode material for Mg-ion batteries.

Moreover, we have calculated the energy barrier for Mg atoms diffusion on monolayer B$_2$C. The mobility of metal-ions defines the rate performance, which is a significant character for electrode materials^39^. Considering the structural asymmetry of B$_2$C monolayer, metal ions can diffuse along three possible paths to the most stable adsorption sites (see insert in Fig. 5). The climbing-image nudge elastic (CI-NEB) calculations based on the diffusion energy profile of the Mg atom are presented in Fig. 5. The maximum barrier energy between the most stable adsorption site for path 1, path 2, and path 3 are calculated to be 0.30, 0.89, and 0.91 eV, respectively. Thus Mg diffusion in B$_2$C shows a strong directional anisotropy. The calculated diffusion barrier is comparable to that of commercially used anode materials based on graphite and TiO$_2$ with a barrier of $0.35-0.65$ eV for Li^40, 41^, indicating that Mg atoms can diffuse easily in B$_2$C monolayer along path 1. In contrast, for the diffusion in the other two cases (path 2 and path 3), rather large barriers mean an unfavorable Mg diffusion along these paths.

Finally, to validate the B$_2$C monolayer as anode material for Mg-ion batteries, ab-initio molecular dynamics (AIMD) simulations were conducted under an NVT (constant number of atoms, volume, and temperature) ensemble at the finite temperature (300 K) to evaluate and check the thermal stability of freestanding B$_2$C. Firstly, the Mg$_{2}$B$_2$C was chosen as the highest Mg concentration to investigate the structural stability of the fully charged B$_2$C monolayer. Figure 6a illustrates an overview of the AIMD simulation results for Mg$_{2}$B$_2$C. The slight fluctuations in the kinetic energy imply the stability of the investigated system at 300 K even after 5 ps. The Mg adatoms were found to be intact and unclustered. Afterward, taking into account the resulting structure from the simulations, the Mg atoms were removed from the B$_2$C monolayer (situation of the fully discharged system), and further AIMD calculations were carried out (see Fig. 6b). The obtained results confirm the stability of the initial pristine structure and prove the reversibility in the charge/discharge process of B$_2$C material.

The structure degeneration (the appearance of various kinds of defects) of anodes is one of the major causes of damage or deterioration of metal-ion batteries. Self-healing materials recently have been shown to improve the cycle life of metal-ion batteries^42^. To examine the susceptibility of B$_2$C material to degradation or self-regeneration, we have created a hypothetical situation in which we induce a hole and check the time-dependent behavior of the material at room temperature (300 K). Single-atom (B or C) was pulled out of the B$_2$C layer creating the defected region. The self-healing process of B$_2$C as a function of time is shown in Fig. 7a and b. The atoms move to the positions of the vacant and heal the system. The final bonds are slightly modified and B$_2$C layer is slightly bulged, nevertheless, the hexagons and rhombi arrangement of atoms is maintained. The self-healing is faster in the case of carbon-defect, however, in both cases, the layers can heal without the Stone-Wales type defects in the final structures of B$_2$C. A successful self-healing is another feature that demonstrates the possibility of practical industrial applications of B$_2$C monolayer to achieve a high-energy electrode for Mg-ion batteries.

## Conclusions

In summary, by using systematic first-principles calculations, the B$_2$C monolayer has been explored as an anode material for the Mg-ion battery for the first time. Combining the binding energy and the degree of deformation of B$_2$C, we conclude that the most stable configuration of the adsorbed Mg ions on the B$_2$C surface is Mg$_{2}$B$_{2}$C. Monolayer B$_2$C keeps the metallic properties after Mg adsorption, which is fundamental for its use as an electrode. Finally, the B$_2$C monolayer exhibits a high theoretical storage capacity of 3187.55 mAh/g. To ensure whether the monolayer B$_{2}$C will not be damaged during the charge/discharge process in rechargeable batteries, AIMD simulations including two layers of Mg that cover both sides of B$_{2}$C at a temperature of 300 K were conducted. The result confirmed that the integral B$_{2}$C structure was well maintained. Considering all these advantages, the B$_2$C monolayer can be regarded as a good candidate for Mg-ion batteries.

## Computational methods

To study electronic properties of investigated materials first-principles calculations are performed within the framework of the density-functional theory (DFT)^43^ as implemented in the Quantum Espresso package^44, 45^. The generalized gradient approximation of Perdew-Burke-Ernzerhof (GGA-PBE) is used for the exchange-correlation functional together with projector-augmented wave (PAW) method. After proper convergence tests we obtained well converged values for the kinetic energy cutoff of the wavefunction equal to 60 Ry and the kinetic energy cutoff for charge density equal to 600 Ry.

The pristine model consists of a $4\times 3$ supercell of B$_2$C (containing 36 atoms). To avoid the interaction between neighboring layers, a vacuum layer of 20 Å in the z-direction is introduced. The van der Waals (vdW) interaction with a DFT-D correction of Grimme was considered^46^. The optimized atomic structures, were obtained by fully relaxing of both atomic positions as well as cell parameters by using the Broyden-Fretcher-Goldfarb-Shanno (BFGS) quasi-Newton algorithm until all forces are smaller than 0.01 eV/Å. The Brillouin zone is sampled utilizing a $36\times 36\times 1$ **k**-mesh in the Monkhorst-Pack scheme. The thermal stability of monolayer B$_2$C was examined by performing ab-initio molecular dynamics (AIMD) simulations at 300 K with a time step of 1 fs using CP2K software^47^.

To visualize the results, we have used the Gnuplot (version 5.4.2)^48^ and Vesta (version 3.5.7) software^49^.

## Data Availability

Correspondence and requests for materials should be addressed to A.P.D.
